# An Accurate Definition of the Status of Inactive Hepatitis B Virus Carrier by a Combination of Biomarkers (FibroTest-ActiTest) and Viral Load

**DOI:** 10.1371/journal.pone.0002573

**Published:** 2008-07-02

**Authors:** Yen Ngo, Yves Benhamou, Vincent Thibault, Patrick Ingiliz, Mona Munteanu, Pascal Lebray, Dominique Thabut, Rachel Morra, Djamila Messous, Frederic Charlotte, Françoise Imbert-Bismut, Dominique Rousselot-Bonnefont, Joseph Moussalli, Vlad Ratziu, Thierry Poynard

**Affiliations:** 1 Service d'Hépato-Gastroentérologie, Groupe Hospitalier Pitié-Salpêtrière, Université Paris VI, CNRS UMR 8149, Paris, France; 2 Laboratoire de Virologie, Groupe Hospitalier Pitié-Salpêtrière, Paris, France; 3 Biopredictive, Paris, France; 4 Fédération de Biochimie, Groupe Hospitalier Pitié-Salpêtrière, Paris, France; 5 Service d'Anatomie Pathologique Groupe Hospitalier Pitié-Salpêtrière, Paris, France; Centre for DNA Fingerprinting and Diagnostics, India

## Abstract

**Background:**

The combination of transaminases (ALT), biopsy, HBeAg and viral load have classically defined the inactive status of carriers of chronic hepatitis B. The use of FibroTest (FT) and ActiTest (AT), biomarkers of fibrosis and necroinflammatory activity, has been previously validated as alternatives to biopsy. We compared the 4-year prognostic value of combining FT-AT and viral load for a better definition of the inactive carrier status.

**Methods and Findings:**

1,300 consecutive CHB patients who had been prospectively followed since 2001 were pre-included. The main endpoint was the absence of liver-related complications, transplantation or death. We used the manufacturers' definitions of normal FT (< = 0.27), normal AT (< = 0.29) and 3 standard classes for viral load. The adjustment factors were age, sex, HBeAg, ethnic origin, alcohol consumption, HIV-Delta-HCV co-infections and treatment.

**Results:**

1,074 patients with baseline FT-AT and viral load were included: 41 years old, 47% African, 27% Asian, 26% Caucasian. At 4 years follow-up, 50 complications occurred (survival without complications 93.4%), 36 deaths occurred (survival 95.0%), including 27 related to HBV (survival 96.1%). The prognostic value of FT was higher than those of viral load or ALT when compared using area under the ROC curves [0.89 (95%CI 0.84–0.93) vs 0.64 (0.55–0.71) vs 0.53 (0.46–0.60) all P<0.001], survival curves and multivariate Cox model [regression coefficient 5.2 (3.5–6.9; P<0.001) vs 0.53 (0.15–0.92; P = 0.007) vs −0.001 (−0.003−0.000;P = 0.052)] respectively. A new definition of inactive carriers was proposed with an algorithm combining “zero” scores for FT-AT (F0 and A0) and viral load classes. This new algorithm provides a 100% negative predictive value for the prediction of liver related complications or death. Among the 275 patients with the classic definition of inactive carrier, 62 (23%) had fibrosis presumed with FT, and 3 died or had complications at 4 year.

**Conclusion:**

In patients with chronic hepatitis B, a combination of FibroTest-ActiTest and viral load testing accurately defined the prognosis and the inactive carrier status.

## Introduction

Finding the best method to evaluate and manage patients infected with the hepatitis B virus (HBV) continues to be a challenge [1]–[3]. The combination of liver biopsy, transaminases (ALT), HBeAg and viral load have classically defined the different statuses of HBV carriers, which are used for patient' management [1]–[4]. This classical definition has limitations related to limitations of these four factors themselves. Furthermore, there is a consensus about the importance of these factors as independent prognostic factors [3], but evidence based data are lacking for their independent prognostic weights. In a recent well-detailed overview, there was not a single longitudinal study that assessed these factors together [3]. Only three longitudinal studies have analyzed the prognostic value of baseline liver fibrosis and activity in 755 patients, but without baseline viral load assessment [5], [6], [7].

Liver biopsy for determining disease grade and stage has limitations (sampling error and observer error) and risks [8], which probably explained the small number of studies with baseline and follow-up biopsies. The appropriateness of repeating biopsy is increasingly questionable, as accurate non-invasive markers have been now validated [8], [9], [10].

Transaminases [4], HBeAg presence or seroconversion and viral load [2], [4], [11], [12], are poor predictors of the severity of liver features, including fibrosis stage and activity grade. Cirrhosis complications, including hepatocellular carcinoma, often occur in patients with HBeAg seroconversion, HBV DNA levels less than 10^4^ copies/ml, or ALT levels between 0.5 and 2 times the upper limit of normal [2]. Therefore, these “classical” criteria are controversial for their influence on therapeutic decisions by clinicians managing HBV carriers, without any assessment of fibrosis stage and activity [2], [4].

Validated noninvasive alternatives to liver biopsy in patients infected with HBV [10] include two combinations of simple serum biochemical markers: FibroTest (FT) (Biopredictive) for the assessment of fibrosis, and ActiTest (AT) (Biopredictive) for the assessment of necroinflammatory activity [13]–[16]. With biopsy as the standard of reference, the diagnostic value of FT for the diagnosis of significant fibrosis (bridging fibrosis), as estimated by the area under the receiver operating characteristics curves (AUROC) in 1,457 patients, is 0.77 (95%CI 0.74 to 0.81) and 0.80 (0.77–0.84) when standardized according to the prevalence of fibrosis stages defining advanced and non advanced fibrosis [17], with better accuracies than ALT [10], [13]–[16].

Because liver biopsy is an imperfect gold standard [18], [19], [20], [21], discordances between FT and biopsy may be related to FT failure and also to biopsy failure. False-positive and false-negative FT results are mainly related to Gilbert syndrome, hemolysis, or acute inflammation [20], [21], [22]. Biomarkers have shown similar or lower error rates than small liver biopsies [20], [21] in patients with chronic hepatitis C. To be useful as alternatives to liver biopsy, noninvasive biomarkers must also demonstrate prognostic value based on hard clinical endpoints: liver disease-related mortality and severe hepatic complications. This has been performed for FT-AT in patients with chronic hepatitis C [21]. The aim of the present study was to similarly validate the prognostic value of FT-AT in patients infected with HBV and to use these non-invasive markers for a simpler definition of HBV inactive carrier status. For these purposes we compared the 4-year prognostic value of combining FT-AT and viral load versus the classic definitions.

## Methods

We hypothesized a) that FT and AT together will help to better define the prognosis of patients with chronic hepatitis B in comparison with viral load, transaminases (ALT) and HBeAg status; b) that the prognostic value of FT will be similar than that of liver biopsy and c) that a combination of FT-AT with viral load will help to better define the status of inactive (“healthy”) carrier versus active carrier.

### Patients

Study patients belonged to a prospective hospital-based cohort of 1,300 patients with chronic hepatitis B infection, seen at our institution from November 2001 to December 2006. For the analysis we identified a “retrospective” subgroup of patients who had been previously studied at our institution before November 2001 and for whom the data was retrospectively gathered.

Inclusion criteria were patients with HBsAg positive for at least 6 months, with an assessment of liver histology done with FT-AT measured in fresh serum and an assessment of viral load performed in the same week. Patients had been referred by general practitioners, private specialists, or public general hospitals, for the staging and treatment of hepatitis B infection. Most patients (91.5%) had no severe complications, and the disease had been discovered by the detection of HBsAg. Liver biopsy was not indicated for all patients. All patients received FT unless the patient refused, or the hospital laboratory was unable to perform the FT. Exclusion criteria were patients with missing FT or viral load, or more than one week between FT and viral load assessments.

Follow-up of patients was performed every 6 months, and FT-AT, viral load or biopsy was repeated as deemed necessary by the physician in charge.

All procedures were followed in accordance with the current revision of the declaration of Helsinki, approved as a non-interventional study by the ethical committee of Groupe Hospitalier Pitié Salpêtrière and all participants gave verbal informed consent. According the French law, in non-interventional study the signed informed consent is not mandatory. Biopsy was performed for routine management of chronic HBV infection and was not related to the study protocol. Consenting patients underwent FT testing if biochemistry unit personnel were available to perform the test and were blinded to the clinical data.

### Biomarkers

FT is a noninvasive blood test that combines the quantitative results of 6 serum biochemical markers, [alpha2-macroglobulin, haptoglobin, gamma glutamyl transpeptidase (GGT), total bilirubin, apolipoprotein A1 and alanine amino transferase (ALT)] with patient age and sex data in a patented artificial intelligence algorithm (USPTO 6,631,330) to generate a measure of fibrosis and necroinflammatory activity in the liver [10]. This method provides a numerical quantitative estimate of liver fibrosis ranging from 0.00 to 1.00, corresponding to the METAVIR scoring system. The FT cutoffs for presumed fibrosis stages were <0.27 METAVIR stage F0, 0.27–0.31 stage F1 (portal fibrosis), 0.32–0.47 (F1–F2), 0.48–0.58 (F2 bridging fibrosis), 0.59–0.73 (F3 many septa) and >0.73 cirrhosis (F4). An algorithm has been suggested that would classify patients into 3 groups: no or minimal fibrosis (FT between 0–0.31), moderate fibrosis (FT between 0.31–0.58), and severe fibrosis (FT between 0.58–1.00) [10].

We measured GGT, ALT, AST, and total bilirubin, with a Hitachi 917 Analyzer and Roche Diagnostics reagents; alpha2-macroglobulin, apolipoprotein A1, and haptoglobin were measured with a BNII (Dade Behring). Personnel blinded to all patient characteristics, including biopsy results, performed all the tests. All the analytical studies, including intra- and interobservers and reproducibility studies, were performed independent of the present study, with CVs <10%, and have been reported previously [10], [22]. There is no perfect definition of normal upper limit for ALT [23]–[24]. Therefore we used the median value 50 IU/L [23] and a more restricted definition at 0.5 times the upper limit that is 25 IU/L as suggested by Lai et al [2].

### Viral load

Blood samples taken at each study visit were subjected to virological analysis without knowledge of the clinical data. Almost all samples were quantified in a single center (GHPS virology department). Only 16/1074 (1.5%) samples were analyzed in another laboratory. HBV serological markers (HBeAg, anti-HBe antibody) were determined using Axsym Abbott's test (Abbott, Les Ulis, FRANCE). From December 2001 to June 2006, serum HBV DNA was measured by PCR using HBV Monitor Cobas (Roche, Meylan, France) with a lower limit of detection of 200 copies/mL. Since June 2006, all samples have been quantified using Cobas Ampliprep Taqman (Roche, Meylan, France) with a lower limit of detection of 12 IU/mL. All HBV viral load quantification units were transformed to International Units according to the manufacturer's specification, i.e. 1 IU/mL equals 5.26 copies/mL as determined with Cobas Monitor.

### Liver biopsy

Liver biopsies were processed with standard techniques. A single pathologist (F.C.), who was unaware of the biochemical markers, evaluated the fibrosis stage and activity grade according to the METAVIR scoring system [25]. Fibrosis was staged on a scale of 0 to 4: F0 = no fibrosis, F1 = portal fibrosis without septa, F2 = few septa, F3 = numerous septa without cirrhosis, F4 = cirrhosis.

### Survival analysis

The prognostic factors were estimated and compared using survival curves, the area under the prognostic ROC curves and multivariate analysis.

#### Endpoints

Only prospective events were analyzed in the main survival analysis. The 4-year survival without HBV-related cirrhosis or liver disease-related complications was the a priori main end-point used to compare the prognostic value of FT versus other biomarkers and histological staging. These complications were defined as: death, liver transplantation, decompensation, variceal bleeding, or hepatocellular carcinoma (HCC), Decompensation was determined by the presence of ascites, hepatic encephalopathy or jaundice (total bilirubin >51 µmol/L). Ascites was deemed to be present when ascitic fluid was confirmed by paracentesis and/or abdominal imaging. HCC was diagnosed by histological examination of liver tissue obtained by liver biopsy, or at autopsy, or if one or more hepatic space–occupying lesions observed at ultrasonography or computed tomography were shown to have vascular patterns typical of HCC by angiography, dual-phase spiral tomography, or magnetic resonance imaging. Variceal bleeding was diagnosed on the basis of endoscopic findings in patients presenting with upper gastrointestinal hemorrhage. Two secondary endpoints were death related to HBV and the overall survival regardless of the cause of death.

#### Survival time

The survival time was calculated from the date of FT to the endpoint date. This interval was censored at 4 years, or if shorter, at the time of last follow-up. For decompensated patients at baseline, only complications occurring during follow-up were taken into account. When several complications occurred, the first one was considered. Each year, for patients who had not been seen at our hospital in the previous 12 months, we found out whether they were living and if not, the date and the cause of death. For patients who were still alive, we either interviewed the patients or obtained information through their physicians. For deceased patients who died outside our hospital, we obtained information about the date and cause of death from their physicians or family. If we could not obtain information on the patient, we sent a letter to the city of their birth in order to find out if they were still living and, if not, the date of death.

#### Prognostic factors

The prognostic value of the biomarkers of liver injury (FT for fibrosis, AT or ALT for activity), and of the previously established seven “important” prognostic factors [3]: three host factors (age, gender, ethnic origin), three virus-related (viral load, HBeAg, coinfection) and one environmental factor (heavy alcohol consumption of 50 g or more per day) were assessed in univariate and multivariate analyses to identify the best combination.

#### Classification of patients

We compared survival of patients classified according to three methods: the baseline usual cutoffs for FT, viral load and ALT, the classical definitions of HBV carriers and the best combination of prognostic factors derived from analyses.

For FT, the previously recommended classification of patients with both hepatitis B and hepatitis C into 3 classes was used [10], [21]: severe (>0.58), intermediate (0.32–0.58) and minimal fibrosis (<0.32). ALT serum activity has been classified [2], [23] in three classes: elevated (50 IU/L or greater), low (25–49 IU/L) and very low (<25 IU/L). The viral load has been classified [1] in three classes: high (>20,000 IU/ml or 10^5^ copies/ml), intermediate (2000–20,000 IU/ml or 10^4^–10^5^ copies/ml) and low (<2000 IU/ml or 10^4^ copies/ml).

The following “classical” definition of HBV carriers was used [1], [3]: 1) Immune tolerance phase: HBeAg positive with high viral load and persistently normal ALT. 2) Immune clearance phase: HBeAg positive with high viral load and elevated ALT. 3) Inactive carrier phase: HBeAg negative, anti-HBe positive, persistently normal ALT. 4) Reactivation phase: HBeAg negative, anti-HBe positive, intermediate or high viral load and elevated ALT.

According to the multivariate analysis, the following new simplified classification was retrospectively assessed: 1) Immune tolerance phase: HBeAg positive with high viral load and normal FT-AT (A0F0). 2) Active phase: HBeAg positive or anti-HBe positive, whatever the viral load, and elevated FT-AT. 3) Inactive carrier phase: HBeAg negative, anti-HBe positive, low or intermediate viral load and normal FT-AT.

The FT prognostic value was compared with that of simultaneous baseline biopsies. We retrospectively compared FT with two other indexes: the classical Child-Pugh score [26], and the APRI index [27].

### Statistical methods

We used the chi-square test for qualitative comparisons, the Mann-Whitney test for quantitative comparisons [28], time-dependent Kaplan-Meier analysis for survival curves, and the log-rank test and Cox proportional hazard model for multivariate analysis [28]. We checked the assumption of proportional hazards by plotting the scaled Schoenfeld residuals [28].

We compared patient survival with the expected survival in the French population, matched for age, sex, and follow-up period. The survival curve of the French population was calculated on the basis of age, sex, and follow-up period and conditional probabilities of death, from official, published census tables [29]. For each patient, beginning from the date of FT assessment, we used the Ederer II method to calculate a yearly predicted cumulative survival rate from a person of the same age and sex having a similar period of follow-up; we used the Z-test for comparisons between actual and predicted survivals [30] to compare the prognostic values of FT and fibrosis staging. FT data, as well as other score values, were entered as continuous variables. We calculated AUROCs with an empirical nonparametric method according to Delong et al [31], or the binormal method if the sample size of the endpoint was <30 [32], and hen compared results with the method of Zhou et al [33]. We used 2-sided statistical tests for all analyses; a p-value of ≤0.05 was considered significant. We used Number Cruncher Statistical Systems 2003 software (NCSS, Kaysville, UT, USA) for all analyses [28].

### Sensitivity analyses

Survival analyses were repeated: after exclusion of patients 1) with FT high-risk profiles of false positives/negatives as identified by security algorithms [20] 2) with clinically obvious cirrhosis. Analyses were also performed according to gender and BMI, in the subgroup of patients without coinfection with Delta, HIV or HCV, and in patients with normal baseline ALT. “Clinically obvious cirrhosis” was defined as a patient with decompensated cirrhosis or with the association of at least two of the following classical signs of cirrhosis: platelets less than 100,000, prothrombin time less than 70%, atrophy of the liver or splenomegaly on ultrasonography, or large varices on endoscopy. The prognostic value of FT was compared with that of elastography (Fibroscan®) when simultaneously performed.

## Results

### Patients

Of the 1,300 patients seen during the study period, 1074 (83%) were included and 226 were non-included (17%) (Table 1). Compared to non-included patients, those included were more often female and less often Caucasian, and has less severe disease (less HIV coinfection, death, less or complications), less HBeAg positive, fewer biopsies, and had received more treatment with anti viral drugs. A total of 9,169 ALT measurements have been performed in included and 1,310 in non-included patients.

**Table 1:** Characteristics of included patients.

	Included Concomitant FT and viral load	Not included	Significance P value
**Number of patients**	**1074**	**226**	
**Characteristics**			
Age at baseline (years) mean (SD)	40.7 (12.6)	41.8 (13.7)	0.24
Male (%)	738 (69)	180 (80)	0.001
Female (%)	336 (31)	46 (20)	
Body Mass Index (kg/m2)	23.9 (4.3)	22.8 (3.9)	0.006
Ethnic origin (%)			0.0005
Caucasian	280 (26)	73 (32)	
African	503 (47)	103 (46)	
Asian	291 (27)	50 (22)	
Source of infection (%)			0.02
Transfusion	53 (4.9)	2 (0.9)	
IV drug	13 (1.2)	3 (1.3)	
Other or unknown	1008 (93.9)	221 (97.8)	
Alcohol (g per day) (%)	(n = 1055)	(n = 220)	0.003
0	869 (82)	166 (75)	
0–50	153 (15)	37 (17)	
Over 50	33 (3)	17 (8)	
At least one co-infection	167 (15.5)	40 (17.7)	0.42
Coinfection HIV (%)	61 (5.7)	22 (9.7)	0.02
Coinfection HCV (%)	62 (5.8)	11 (4.9)	0.59
Coinfection Delta	75 (7.0)	15 (6.6)	0.85
Baseline viral load (KUI/ml) (SD)	4462 (56462) (n = 1074)	10281 (26994) (n = 203)	0.15
Low <200 IU/ml	683 (63.6%)	89 (43.8%)	P<0.001
Intermediate 200–20,000 IU/ml	169 (15.7%)	15 (7.4%)	
High >20,000 IU/ml	222 (20.7%)	99 (48.8%)	
**Risk factors**			
Diabetes (%)	44 (4.1)	9 (4.0)	0.94
Renal failure (%)	34 (3.2)	9 (4.0)	0.53
**Simultaneous biopsies (n)**	**97**	**62**	
Advanced fibrosis F2F3F4 (%)	33 (33%)	32 (52%)	0.03
Moderate-severe activity A2A3 (%)	19 (20%)	26 (43%)	0.002
Steatosis >5% (%)	47/97 (49)	19/62 (31)	0.03
**All biopsies (n)**	**505**	**132**	
Advanced fibrosis F2F3F4 (%)	191 (38%)	60 (46%)	0.11
Moderate-severe activity A2A3 (%)	144 (29%)	47 (36%)	0.11
Steatosis >5% (%)	212/434 (49%)	49/104 (47%)	0.75
**Biomarkers (SD)**			
Number performed	1074	0	
ALT UI/L	80 (270) (n = 1074)	126 (283) (n = 226)	0.02
Total Bilirubin umoles/L	19.9 (53.3) (n = 1074)	39.9 (103.7) (n = 98)	0.001
GGT IU/L	52.3 (95.8) (n = 1074)	106.4 (159.4) (n = 94)	<0.001
Alpha2 macroglobulin g/L	2.22 (0.76)	NP	
ApoA1 g/L	1.45 (0.35)	NP	
Haptoglobin g/l	0.87 (0.55)	NP	
FibroTest (0.00–1.00)	0.34 (0.26)	NP	
ActiTest (0.00–1.00)	0.26 (0.25)	NP	
Duration prospective follow-up	2.5 (0.5)	4.8 (0.3)	
Duration retrospective follow-up	5.2 (0.2)	3.0 (0.4)	
Treated for HBV	646 (60%)	102 (45%)	<0.001
Complications retrospective follow-up	87 (8.1%)	23 (10.2%)	0.30
Complications (not lethal) prospective follow-up	14 (1.3%)	29 (12.8%)	<0.001
Death related to HBV	27 (2.5%)	23 (10.2%)	<0.001
Death or complications related to HBV	41 (3.8%)	52 (23.0%)	<0.001
Death not related to HBV	9 (0.8%)	7 (3.1%)	0.005
Overall death	36 (3.4%)	30 (13.3%)	<0.001

NP = Not performed.

### Survivals

Among the 1,074 included patients with baseline FT-AT and viral loads, the mean follow-up was 7.7 years (2.5 years prospective and 5.2 retrospective); after 4 years of prospective follow-up, 50 complications occurred (survival without complications 93.4%), and 36 deaths (survival 95.0%), including 27 related to HBV (survival 96.1%) (Table 2, Table 3). The number of patients still at risk in the prospective follow-up was 655 at 2 years, and 242 at 4 years.

**Table 2:** Causes of death and complications during the 4-year follow-up.

Death related to HBV N = 27	Complications without death N = 14	Death not attributable to HBV N = 9
Hepatocellular carcinoma n = 19 (including 3 with hemorrhage)	Hepatocellular carcinoma n = 6 (1 transplanted)	Non Liver cancer: n = 2;
Hemorrhage n = 4	Hemorrhage n = 2 (1 transplanted)	Neurologic: n = 3; Cardiac: n = 2; Accident: n = 1;
Decompensation n = 4 (including cirrhosis n = 1, reactivation n = 2 and post transplantation n = 1)	Decompensation n = 6	Unknown: n = 1

**Table 3:** 4-year survival according to baseline FibroTest, viral load ALT values and treatment.

Baseline FibroTest Value	n	Death or HBV complications	Survival without HBV complications	HBVRelated Death	Survival without HBV death	Death	Overall Survival	Overall Survival in paired controls
**0.00–0.31**								
No or minimal fibrosis	637	4	98.9 (97.7–100)	1	99.4 (98.4–100)	1	99.4 (98.4–100)	99.5 (99.5–99.6)
Not treated	350	0	100	0	100	0	100	99.6 (99.5–99.6)
Treated	287	4	98.0 (96.1–99.9)	1	99.2 (97.5–100)	1	99.2 (97.5–100)	99.5 (99.4–99.6)
**0.32–0.58**								
Moderate fibrosis	229	6	94.1 (88.8–99.5)	1	99.4 (98.3–100)	3	98.2 (96.2–100)	98.5 (98.0–98.9)
Not treated	54	1	95.2 (86.1–100)	0	100	1	95.2 (86.1–100)	98.2 (97.0–99.3)
Treated	175	5	93.9 (87.9–99.9)	1	99.3 (98.0–100)	2	98.7 (96.9–100)	98.6 (98.1–99.0)
**0.59–1.00**								
Severe fibrosis§	208	40	77.6 (71.3–83.9)*	25	84.2 (77.9–90.5)	32	80.5 (73.8–87.1)§§	97.3 (96.7–97.9)
Not treated	24	7	70.0 (51.4–88.6)	6	74.1 (56.2–92.0)$	7	70.0 (51.4–88.6)£	97.1 (94.6–99.5)
Treated	184	33	78.7 (71.9–85.4)	19	87.5 (81.8–93.1)	25	81.8 (74.8–88.9)	97.3 (96.7–97.9)
**Viral load** ***								
Low <2000 IU/ml	683	24	94.7 (92.3–97.0)	12	97.5 (96.0–99.0)	18	96.2 (94.4–98.1)	98.8 (98.6–99.1)
Not treated	332	6	97.4 (95.2–99.5)	4	98.5 (97.0–99.9)	6	97.4 (95.2–99.5)	99.1 (98.9–99.4)
Treated	351	18	93.2 (89.9–96.5)	8	97.0 (94.9–99.2)	12	95.6 (93.1–98.2)	98.5 (98.3–98.8)
Intermediate 2000–20,000 IU/ml	169	2	98.8 (97.1–100)	0	100	1	99.4 (98.1–100)	99.0 (98.6–99.5)
Not treated	61	0	100	0	100	0	100	99.6 (99.5–99.7)
Treated	108	2	98.1 (95.6–100)	0	100	1	99.1 (97.2–100)	98.7 (97.9–99.5)
High >20,000****	222	24	85.4 (79.4–91.4)	15	89.2 (82.9–95.5)	17	88.2 (81.8–94.5)	98.9 (98.5–99.2)
Not treated	35	2	92.5 (82.3–100)	2	92.5 (82.3–100)	2	92.5 (82.3–100)	99.6 (99.4–99.8)
Treated	187	22	84.5 (78.0–91.1)	13	88.8 (81.9–95.8)	15	87.7 (80.6–94.7)	98.7 (98.3–99.1)
**ALT**								
Very Low <25 IU/L	317	5	97.9 (96.0–99.8)	3	98.9 (97.6–100)	5	97.9 (95.9–99.8)	98.9 (98.7–99.2)
Not treated	176	1	99.4 (98.3–100)	1	99.4 (98.3–100)	1	99.4 (98.3–100)	99.3 (98.9–99.6)
Treated	141	4	96.6 (93.3–99.9)	2	98.4 (96.3–100)	4	96.6 (93.3–99.9)	98.5 (97.9–98.9)
Low 25–49 IU/L	455	16	94.0 (90.5–97.5)	3	99.2 (98.2–100)	8	97.7 (96.1–99.3)	98.9 (98.7–99.2)
Not treated	205	3	97.4 (94.4–100)	1	99.3 (97.9–100)	3	97.4 (94.4–100)	99.2 (98.9–99.5)
Treated	250	13	92.4 (87.6–97.1)	2	99.1 (97.9–100)	5	97.8 (95.9–99.7)	98.7 (98.3–99.1)
Elevated > = 50 IU/L	302	29	87.9 (83.6–92.2)	21	89.7 (85.0–94.4)	23	89.0 (84.2–93.8)*$	98.7 (98.4–99.0)
Not treated	47	4	88.9 (78.5–99.2)	4	88.9 (78.5–99.2)	4	88.9 (78.5–99.2)	99.0 (98.2–99.9)
Treated	255	25	87.8 (83.2–92.5)	17	89.9 (84.8–95.1)	19	89.1 (83.9–94.3)	98.6 (98.3–98.9)
**All**	**1074**	**50**	**93.4 (91.4–95.4)**	**27**	**96.1 (94.4–97.8)**	**36**	95.0 (93.2–96.8)*	**98.9 (98.7–99.0)**

§ Survival of the severe fibrosis group was significantly lower than the two other groups (P<0.001).

§§ Overall survival of the severe fibrosis group, treated or not, was significantly lower than that of paired controls (p<0.05).

* Overall survival of the 1074 HBV patients, was significantly lower than that of paired controls (p<0.05).

$ P = 0.03 vs treated.

£ P = 0.047 vs treated.

*** Survivals of the treated patients were similar to those of the non –treated in different groups of viral load (p>0.05).

**** Overall survival of the group with high viral load was lower than that of paired controls (p<0.05).

$* Overall survival of the group with elevated ALT was lower than that of paired controls (p<0.05).

We used the manufacturers' definitions of normal FT (< = 0.27), normal AT (< = 0.29) and 3 classes for viral load in IU/ml.

### Biomarkers and viral load

A total of 2,573 FT-AT and 1,597 viral load tests were assessed; biopsy was performed at least once in 505 patients, and at baseline simultaneously with viral load testing and FT-AT in 97 patients and elastography in 270 patients.

### Treatment of chronic hepatitis B

A total of 646 patients have been treated (mean of 1.6 different treatments per patient), 97 with interferon, 78 with pegylated interferon, 552 with lamivudine, 247 with adefovir, 55 with tenofovir and 67 with entecavir. Patients were treated according to standard guidelines (ALT or biopsy), and more recently according to new evolving criteria: viral load, FT or Fibroscan. The distribution of these criteria were: elevated ALT (n = 255), F1 or greater at biopsy (n = 295), A1 or greater at biopsy (n = 304), DNA equal or greater than Log5 (n = 187), FT greater than F0 (n = 447), ActiTest greater than A0 (n = 407), liver stiffness measurements greater than 7.1 kPa (n = 80). A total of 508 patients (79%) have been treated according to standard criteria (ALT, biopsy and viral load) and 138 (21%) according to FT-AT, Fibroscan or specific protocols.

### Accuracy of biomarkers for the diagnosis of advanced fibrosis

The accuracy of FT for the diagnosis of advanced fibrosis was compared with other available markers. It was similar to previous validations, and higher than ALT, viral load and APRI, both using simultaneous biopsies (n = 97, AUROC = 0.83 95%CI 0.71–0.91) versus ALT (0.60; 95%CI 0.47–0.71 P = 0.0007), viral load (0.55; 95%CI 0.42–0.66 P = 0.0002) and APRI index (0.66; 95%CI 0.51–0.77 P = 0.002); or in all biopsies (n = 505, AUROC = 0.78 95%CI 0.73–0.82) versus ALT (AUROC = 0.57; 0.50–0.62 P<0.001), viral load (AUROC = 0.53; 0.47–0.58 P<0.001) and APRI index (0.57; 95%CI 0.51–0.63 P<0.001).

### Prognostic values of biomarkers viral load and biopsy

#### Survival curves

The survival outcomes of patients classified according to previously defined FT cut-offs are presented in Table 3. Survivals of the treated patients were similar to those of the non–treated in different groups of viral load (Table 3).

In the minimal severity group there were 4 complications: 3 patients with hepatocellular carcinoma (including one death), without cirrhosis, and one patient without cirrhosis treated with lamivudine with one flare-up associated with viral resistance, i.e. 98.9% (95%CI, 97.8%–100%) survival without complications.

In the moderate group, there were 6 complications, including one HBV – related death, i.e. 94.1% (95%CI, 88.8%–99.5%) survival without complications.

In the severe group there were 40 complications including 25 HBV- related deaths, i.e. 77.6% (95%CI, 71.3%–83.9%) survival without complications. Survivals of the treated patients were higher than those of the non –treated in patients with severe fibrosis (Table 3).

#### Area under the prognosis ROC curves

Among the 1,074 patients the prognostic AUROCs of FT were greater than AT, ALT or viral load for all the prognostic criteria (Table 4). For the main endpoint, survival without complications, AUROCs were 0.89 (0.84–0.93) vs 0.77 (0.69–0.83), 0.53 (0.46–0.60) and 0.64 (0.55–0.71), respectively (all P<0.004).

**Table 4:** Comparison of Area under the Receiver Operating Characteristics Curves (AUROC) for survival endpoints, between FibroTest, ActiTest, ALT and viral load. N = 1074.

Marker	Number of patients	Survival without HBV complications		Survival without HBV death		Overall Survival	
		**AUROC**	**95% CI**	**AUROC**	**95% CI**	**AUROC**	**95% CI**
**FibroTest**	1074	0.89	0.84–0.93^c^	0.95	0.91–0.97 d	0.94	0.89–96^e^
Non-treated	428	0.99	0.89–0.90	0.99	0.96–0.99	0.99	0.89–0.99
Treated	646	0.82	0.75–0.88	0.90	0.84–0.94	0.89	0.83–0.93
**ActiTest**	1074	0.77	0.69–0.83	0.87	0.79–0.92	0.81	0.72–0.87
Non-treated	428	0.86	0.58–0.96	0.91	0.57–0.98	0.86	0.58–0.96
Treated	646	0.70	0.61–0.77	0.83	0.73–0.89	0.76	0.65–0.84
**ALT**	1074	0.53	0.46–0.60	0.55	0.44–0.65	0.55	0.46–0.63
Non-treated	428	0.54	0.46–0.62	0.57	0.48–0.64	0.54	0.46–0.62
Treated	646	0.51	0.44–0.58	0.54	0.42–0.64	0.53	0.44–0.62
**Viral load**	1074	0.64	0.55–0.71	0.67	0.54–0.76	0.63	0.52–0.72
Non-treated	428	0.57	0.30–0.76	0.62	0.28–0.82	0.57	0.29–0.76
Treated	646	0.61	0.51–0.69	0.65	0.51–0.76	0.61	0.49–0.71

c FibroTest AUROC greater than that with ActiTest (p<0.001), ALT (p<0.001), Viral load (p<0.001).

d FibroTest AUROC greater than that with ActiTest (p = 0.0009), ALT (p = <0.001), Viral load (p<0.001).

e FibroTest AUROC greater than that with ActiTest (p<0.001), ALT (p<0.001), Viral load (p<0.001).

The AUROC of FT versus fibrosis staging at biopsy for the prognosis or HBV-related death or complications were similar among the 97 patients with simultaneous assessments at baseline: 0.98 (0.89–0.99) versus 0.97 (0.93–0.99; P = 0.71) (Table 5).

**Table 5:** Comparison of Area under the Receiver Operating Characteristics Curves (AUROC) for survival endpoints, between FibroTest and simultaneous histology. N = 97.

Marker	Number of patients	Survival without HBV complications		Survival without HBV death		Overall Survival	
		**AUROC**	**95% CI**	**AUROC**	**95% CI**	**AUROC**	**95% CI**
**FibroTest**	97	0.98	0.89–0.99^a^	0.96	0.85–0.99 a	0.98	0.89–0.99 a
Non-treated	26	1.00		NP b		1.00	
Treated	71	0.96	0.84–0.99	0.96	0.84–0.99	0.96	0.84–0.99
**Fibrosis Staging at biopsy**	97	0.97	0.93–0.99	0.96	0.93–0.98	0.97	0.93–0.99
Non-treated	26	0.98	0.86–0.99	NP b		0.98	0.86–0.99
Treated	71	0.96	0.92–0.98	0.96	0.92–0.98	0.96	0.92–0.98

a FibroTest AUROC was similar to that with fibrosis staging.

b NP, not performed because the number of events was too low.

The FT AUROCs for survival endpoints were also all greater (P<0.01) than the AUROCs of the other indexes, the Pugh, and APRI (Table 6).

**Table 6:** Comparison of Area under the Receiver Operating Characteristics Curves (AUROC) for survival endpoints, between FibroTest, ActiTest, ALT, viral load, Pugh score and APRI index. N = 978.

Biomarker	Number of patients	Survival without HBV complications f		Survival without HBV death g		Overall Survival h	
	**978**	**AUROC**	**95% CI**	**AUROC**	**95% CI**	**AUROC**	**95% CI**
**FibroTest**	978	0.89	0.83–0.93	0.95	0.91–0.97	0.94	0.89–0.96
Non-treated	388	0.98	0.83–0.99	0.99	0.92–0.99	0.98	0.83–0.99
Treated	590	0.82	0.74–0.87	0.90	0.84–0.94	0.89	0.83–0.93
**ActiTest**	978	0.77	0.69–0.84	0.87	0.79–0.93	0.81	0.72–0.88
Non-treated	388	0.84	0.51–0.95	0.89	0.45–0.98	0.84	0.51–0.95
Treated	590	0.71	0.62–0.79	0.83	0.74–0.89	0.77	0.67–0.85
**ALT**	978	0.54	0.46–0.62	0.56	0.44–0.66	0.56	0.46–0.65
Non-treated	388	0.62	0.43–0.75	0.67	0.43–0.82	0.62	0.43–0.75
Treated	590	0.52	0.44–0.59	0.54	0.42–0.65	0.54	0.44–0.63
**Viral Load**	978	0.66	0.57–0.74	0.68	0.55–0.78	0.64	0.53–0.73
Non-treated	388	0.59	0.29–0.78	0.65	0.26–0.86	0.59	0.29–0.78
Treated	590	0.64	0.54–0.72	0.65	0.51–0.76	0.63	0.50–0.73
**Pugh classification**	978	0.82	0.72–0.89	0.89	0.76–0.95	0.87	0.75–0.93
Non-treated	388	0.92	0.51–0.98	0.97	0.35–0.99	0.92	0.51–0.98
Treated	590	0.79	0.67–0.87	0.86	0.69–0.94	0.84	0.70–0.92
**APRI Index**	978	0.55	0.49–0.61	0.58	0.49–0.67	0.57	0.49–0.63
Non-treated	388	0.66	0.28–0.86	0.68	0.21–0.89	0.66	0.28–0.86
Treated	590	0.53	0.47–0.58	0.55	0.47–0.63	0.54	0.47–0.60

f FibroTest AUROC greater than that with ActiTest (p = 0.001), ALT (p<0.001), Viral Load (p<0.001), Pugh classification (p = 0.0025), APRI Index (p<0.001).

g FibroTest AUROC greater than that with ActiTest (p = 0.0016), ALT (p<0.001), Viral Load (p<0.001), Pugh classification (p = 0.005), APRI Index (p<0.001).

h FibroTest AUROC greater than that with ActiTest (p<0.001), ALT (p<0.001), Viral Load (p<0.001), Pugh classification (p = 0.002), APRI Index (p<0.001).

#### Multivariate prognostic analysis

In the prospective follow-up, FT-AT, age, male gender, Caucasian origin, viral load and heavy alcohol consumption were associated with survival without complications or death in univariate analysis. In multivariate analysis FT (P<0.001) and viral load (P = 0.007) were the most significant independent factors, with a marginal significance (P = 0.052) for ALT, and age (P = 0.03) (Table 7). HBV treatment has no significant impact on survival when adjusted on FT, viral load and age.

**Table 7:** Prognostic factors associated with survival without HBV complications or death in 1074 patients.

Baseline factor	Univariate			Multivariate		
	Regression coefficient	95% CI	P value	Regression coefficient	95% CI	P value
**Biomarker liver injury**						
FibroTest	5.42	4.24–6.61	<0.001	5.21	3.53–6.88	<0.001
ActiTest	2.84	2.01–3.67	<0.001	0.41	−1.03–1.84	0.581
ALT	0.0003	−0.0004∶0.001	0.49	−0.001	−0.003−0.000	0.052
**Host factor**						
Older age	0.06	0.04–0.08	<0.001	0.026	0.003–0.048	0.026
Male gender	1.41	0.49–2.34	0.003	0.55	−0.42–1.52	0.266
Caucasian	0.75	0.20–1.31	0.008	0.07	−0.56–0.69	0.827
**Viral related**						
Viral load	0.57	0.26–0.87	0.0003	0.53	0.15–0.92	0.007
HbeAg	0.58	−0.03–1.20	0.06	0.13	−0.64–0.88	0.746
Coinfection HCV, HIV or Delta	0.45	−0.24–1.14	0.20	−0.29	−1.12–0.53	0.478
**Other factor**						
Alcohol consumption > = 50 g/day	1.78	1.08–2.47	<0.001	0.67	−0.10–1.44	0.091
**Treatment effect**	0.99	0.23–0.75	0.01	−0.25	−1.09–0.59	0.563

In the retrospective follow-up, only FT was associated with HBV complications [(logistic regression coefficient =  6.2; 95%CI 5.0–7.4 (P<0.001)].

### Definition of inactive HBV carrier

The classical definition of inactive carrier was observed in 275 untreated patients, without coinfection with HCV, Delta or HIV. The negative predictive value of this definition was 98.1% at 4 years; 3 patients died, one of HBV (decompensated cirrhosis, presumed at FT and confirmed at biopsy), another patient died from lung cancer (with non-decompensated cirrhosis, presumed at FT and confirmed with biopsy) and the last from an unknown cause; 62 (23%) of these “classically defined inactive carrier” patients had fibrosis presumed with FT: 13 F1 (portal fibrosis), 31 F1–F2, 9 F2 (bridging fibrosis), 4 many septa (F3) and 5 cirrhosis (F4).

Among these 275 classical inactive carriers, none had viral coinfection, 11 (4%) were heavy drinkers, and none had hepatocellular carcinoma. Among these 11 drinkers, 3 had fibrosis. When these 11 patients were excluded, the percentage of pure HBsAg carrier with abnormal FTs indicating fibrosis was still high = 22% (59/264).

**Figure 1:**
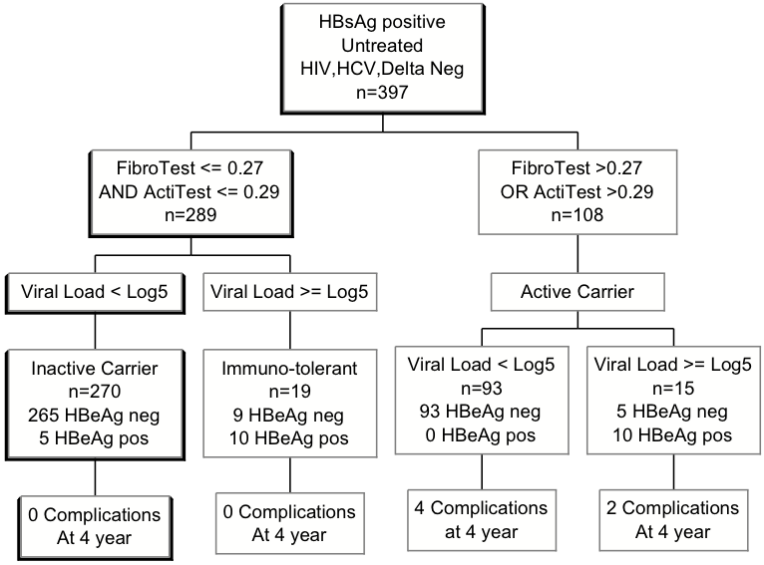
Untreated patients classified according to biomarkers, viral load and HBeAg No liver related complications occurred during the 4-year follow-up among patients with baseline normal FibroTest and normal ActiTest. This new definition had a 100% negative predictive value for liver related complications or death. Using the classical definition of inactive carrier with normal transaminases, 23% had presumed fibrosis, and 3 complications occurred during the follow-up.

A new definition of inactive carriers (n = 289, untreated patients, without coinfection with HCV, Delta or HIV) was proposed with an algorithm combining “zero” scores for FT-AT (F0 and A0) and different viral load levels (Figure 1). This new algorithm provides a 100% NPV for the prediction of liver related complications, both in HBeAg negative and positive cases.

The comparisons of survival curves between these inactive carriers defined with normal FT-AT (F0A0) versus active carriers (nonF0A0) are detailed in Table 8.

**Table 8:** Survival according to definition of inactive carrier based on FibroTest-ActiTest normal values in untreated patients.

Baseline definition of inactive carrier	N	HBV complications	Survival without HBVcomplications	HBV Related Death	Survival without HBV death	Death	Overall Survival	Overall Survival in paired controls
**All untreated patient**	428	8	97.4 (95.5–99.2)	6	98.2 (96.8–99.7)	8	97.4 (95.5–99.2)	99.2 (99.0–99.5)
**Normal FibroTest and ActiTest** *	303	0	100**	0	100**	0	100**	99.6 (99.5–99.6)
**Abnormal FibroTest or ActiTest** ***	125	8	90.8 (84.5–97.1)	6	93.7 (88.8–98.7)	8	90.8 (84.5–97.1)	98.5 (97.8–99.2)
**Without HIV, HCV, Delta**	397	6	97.7 (95.8–99.6)	4	98.7 (97.3–99.9)	6	97.7 (95.8–99.6)	99.2 (99.0–99.5)
Normal FibroTest and ActiTest	289	0	100	0	100	0	100	99.6 (99.5–99.6)
Abnormal FibroTest or ActiTest	108	6	91.2 (84.2–98.1)	4	94.7 (89.7–99.8)	6	91.2 (84.2–98.1)	98.4 (97.6–99.1)
**With HIV, HCV, Delta** ***	31	2	92.8 (83.2–100)	2	92.8 (83.2–100)	2	92.8 (83.2–100)	99.4 (99.2–99.6)
Normal FibroTest and ActiTest	14	0	100	0	100	0	100	99.5 (99.2–99.7)
Abnormal FibroTest or ActiTest	17	2	87.5 (71.3–100)	2	87.5 (71.3–100)	2	87.5 (71.3–100)	99.3 (99.0–99.7)
**Classical Inactive carrier ** $								
Yes	275	3	98.1 (95.9–100)	1	99.5 (98.5–100)	3	98.1 (95.9–100)	99.1 (98.8–99.4)
No	153	5	95.9 (92.4–99.5)	5	95.9 (92.4–99.5)	5	95.9 (92.4–99.5)	99.4 (99.2–99.7)

* Both normal values: FibroTest < = 0.27 and ActiTest < = 0.29.

** Survivals of patients with abnormal FibroTest and ActiTest were lower than those of normal FibroTest and ActiTest (p<0.005).

*** Survivals of patients with or without one coinfection (HVI, HCV, Delta) were not significantly different (p>0.05).

**** Overall survivals of patients with abnormal FibroTest and ActiTest were lower to those in paired controls (p<0.005).

$ Inactive carrier: anti HBe, normal ALT, viral load <2,000 IU/ml, no coinfection HCV, HIV, or Delta.

Thus we have proposed three categories of F0A0 according to viral load (Figure 1).

The first is the inactive carrier category with low or intermediate viral load; 270 (93.4%) patients A0F0 belonged to this category including 265 HBeAg negative and 5 HBeAg positive patients.

The second is the category of immuno-tolerant HBeAg positive patients with high viral load: 19 (3.5%) patients A0F0 belonged to this category.

The third category is HBeAg negative and anti-HBe positive patients with high viral load: nine (3.1%) patients A0F0 belonged to this category.

### Repeated biomarkers in inactive carriers

FT-AT was repeated during follow-up in 160 inactive carriers with excellent reproducibility. The Spearman correlation coefficients were 0.74, 0.64, 0.77 between baseline and the second, third and fourth assessments respectively for FT (P<0.001). Only one patient (1/160 = 0.6%) had a repeated FT suggesting advanced fibrosis (FT = 0.49), with a high-risk profile of false positive (hemolysis suspected with haptoglobin = 0.24 g/L and unconjugated bilirubin = 32 microm/L).

For AT the correlations were 0.67, 0.66, 0.67 between baseline and the second, third and fourth assessments, respectively (P<0.001). Only two patients (2/160 = 1.2%) had a repeated AT suggesting moderate activity (AT = 0.53, 0.54). One of these two patients was an HBeAg negative patient, with high baseline viral load and heavy alcohol consumption (A1F0 at biopsy).

For ALT the correlations were 0.65, 0.63, 0.63 between baseline and the second, third and fourth assessments, respectively (P<0.001).

Among 289 patients with baseline normal ALT, 277 (93.4%) had a repeated normal ALT for 18 months or more.

### FibroTest failure

Security algorithms identified 16/1074 (1.5%) patients with high risk profiles of false positives/negatives: 11 with very low haptoglobin concentration (hemolysis or anhaptoglobinemia), 3 with suspected Gilbert's syndrome, and 2 with very low apolipoprotein A1 concentration, which could have a significant impact (0.30 or more) on the FT score.

The FT-AT AUROCs predictive values for survival without HBV complications were still highly significant after exclusion of 16 patients with FT high-risk profiles of false positives/negatives (n = 1,058 AUROC = 0.88, 95%CI 0.81–0.92; P<0.001),

### Impact of HBV treatment on biomarkers

Among the treated patients, the first FT has been performed in 276 patients before treatment and among 370 patients during or after treatment.

Among the 213 treated patients the repeated FT decreased from a mean baseline 0.34 (95%CI 0.30–0.37) to 0.29 (95% CI 0.26–0.32; P<0.001) and the AT from 0.34 (95%CI 0.30–0.38) to 0.19 (95%CI 0.17–0.21; P<0.001). The impact of treatment on biomarkers was higher among the 95 patients with baseline moderate or severe fibrosis: the FT decreased from a mean baseline 0.56 (95%CI 0.52–0.59) to 0.45 (95% CI 0.40–0.49; P<0.001) and the AT from 0.47 (95%CI 0.41–0.53) to 0.23 (95%CI 0.20–0.27; P<0.001).

### Sensitivity analyses

#### Confounding factors

The FT prognostic value was very significant both among males using prognostic AUROC for survival without complications = 0.85 95%CI = 0.77–0.90 as well as in females (AUROC = 0.94 95%CI 0.83–0.98). The FT prognostic value was very significant both among 555 patients with BMI lower than 27 kg/m^2^ using prognostic AUROC for survival without complications = 0.85 95%CI = 0.73–0.91 as well as in 146 patients with BMI > = 27 kg/m^2^ (AUROC = 0.96 95%CI 0.89–0.98).

#### Clinically obvious cirrhosis

The FT-AT AUROCs predictive values for survival without HBV complications were still highly significant after exclusion of 47 (4.4%) patients with clinically obvious cirrhosis (n = 1047 AUROC = 0.82, 95%CI 0.71–0.88; P<0.001)

#### Patients with coinfection with Delta, HIV or HCV

The FT-AT AUROCs predictive values for survival without HBV complications were still highly significant and after exclusion of 167 patients without coinfection with Delta, HIV or HCV (n = 907, AUROC = 0.87, 95%CI 0.80–0.92; P<0.001). They were significant among patients with coinfection (n = 167, AUROC = 0.85, 95%CI 0.76–0.91), and in patients with normal baseline ALT (n = 772, AUROC = 0.87 95%CI 0.81–0.94) or elevated ALT (n = 302, AUROC = 0.82, 95%CI 0.71–0.89). The group of 14 patients coinfected with HCV, Delta or HIV and normal FT-AT had also no death or complications at 4 years versus 2 among the 17 patients with abnormal FT-AT.

#### Patients with elastography

Among 270 patients who had both FT and elastography, the prognostic AUROCs were 0.80, 95%CI 0.55–0.92 and 0.71 95%CI 0.40–0.87; P = 0.42, respectively. There was a very significant concordance between FT and liver stiffness measurements (Spearman correlation = 0.47; P<0.001).

## Discussion

Among the three working hypotheses, all were confirmed. FT and AT together with viral load helped to better define the prognosis of patients with chronic hepatitis B compared to viral load, transaminases ALT and HBeAg status. The prognostic value of FT was similar to that of liver biopsy when simultaneously performed, as observed in patients with chronic hepatitis C [21]. A simple combination of FT-AT with viral load assessment has helped to better define the status of inactive (“healthy”) carriers versus active carriers.

Until now, the prognostic markers recommended for use in chronic HBV, relied on histological fibrosis staging of biopsy specimens, viral load, and HBeAg status, have never been assessed altogether in a longitudinal study on a large number of patients [1]–[3], [34]–[35].

### Limitations of the study

Our study has several limitations.

#### Tertiary center bias

The included population was not a random, community-based population. In a tertiary center the major risk of bias is an over-representation of patients with severe disease. However the characteristics of included patients were similar to published studies on global populations [1]–[3] (Table 1). There was no over-representation of severe diseases with only one-third of patients with advanced fibrosis diagnosed using biopsy or FT and 40% of patients never treated. Clinically obvious cirrhosis at baseline represented only 4% of included patients. Sensitivity analyses excluding these patients gave similar results. A possible bias in European studies can be the non-inclusion of non-Caucasian patients. In the present cohort Caucasians and North Africans represented only 26% of the population and the prognostic value of FT persisted after adjustment for the ethnic origins.

#### Study power

We acknowledge that the number of events was small for death and complications among the inactive carrier, but the number of patients with fibrosis missed by the classical definition of inactive carrier (62 patients with fibrosis, that is 23% of the so called “inactive carrier”) was impressive. As there is an obvious rational relationship between the presence of fibrosis, and the possible progression to cirrhosis and to severe complications, the message for patients and clinicians is important. The negative predictive value of the classical definition is not sufficient to exclude advanced fibrosis and therefore to exclude a risk of complications even during a 4 year follow-up. At least 18 patients out of these “inactive carrier” had bridging fibrosis, many septa or cirrhosis, and needed a treatment to prevent complications. For patients with portal fibrosis or intermediate stage between portal and bridging fibrosis the treatment is less urgent, but they should be monitored more frequently than patients without fibrosis.”

We acknowledge that the number of patients with simultaneous biopsy and FT-AT (n = 97) was small. However FT-AT have been previously extensively validated in patients with chronic hepatitis B (n = 1,457), whether treated or not [10], [13]–[16]. A new validation was also performed in the present study on a total of 505 patients with similar accuracy, both in the simultaneous or non-simultaneous biopsies. It seems very difficult nowadays to convince a large group of patients of the utility of first line liver biopsy, particularly in patients with non-elevated transaminases.

The number of patients with profiles of immunotolerance was small and further validations are needed in such populations [36].

Another limitation of our study is the limited number of patients (n = 61) with coinfection with HIV, as indinavir and atazanavir, can increased significantly unconjugated bilirubin, with a risk of FT false positive. However our results confirmed that in patients with coinfection with HBV or HCV and HIV, the FT diagnostic value was similar than in mono infected patients [37]. No false positives have been observed among the nine patients treated with these two drugs.

### Advantages of the study

#### Prognostic value

Our results indicate that the combination of FT together with baseline viral load was the best combination for predicting survival without complications at 4-years, regardless of the treatment and other risk factors.

The overall survival of patients with non-severe fibrosis at baseline was close to that of paired controls in the general population. In patients with severe fibrosis, overall survival was 17% lower than that of the control population. In patients without clinically obvious cirrhosis the overall survival was better, but still lower than in the control population.

#### Prognostic factors

We have assessed the independent prognostic values of the most important identified prognostic factors [3]. After taking into account FT and viral load, only age had marginal prognostic values. Their respective associations with fibrosis probably confounded the univariate prognostic value of male gender, alcohol consumption and ethnic origin. We acknowledge that we focused on the most important prognostic factors [3]. Future studies should also include HBV genotype assessment, metabolic factors as well as the presence of liver steatosis.

#### Comparison with other prognostic markers

Despite a highly significant difference in favor of FT, the Pugh score prognostic value was good. However, for clinicians the advantage of FT is to have a consistent prognostic value from early fibrosis stage to cirrhosis.

Although this prognostic study was not specifically designed to validate FT as a true surrogate endpoint of the severity of HBV chronic hepatitis [38], we observed as in HCV chronic hepatitis that FT fulfilled almost all of the 13 criteria of a surrogate endpoint biomarker [21], [39], including specificity and sensitivity for fibrosis [10], [20]–[22]. FT is indicative of the response to HBV virological treatment, with FT improvement and cirrhosis reversal [14], [16]; Intra and interobserver variability of FT has been studied; preanalytical and analytical recommendations have also been issued [10], [20]–[22]. Serial monitoring of FT is possible [14], [16], [21], [40], [41], [42]. In comparison, liver biopsy does not satisfy several quality criteria as a surrogate endpoint marker [39].

Our data confirm that FT has a better prognostic value than ALT, even using a very low definition of normal upper limit, or APRI. This was expected since these indexes have lower values for the diagnosis of advanced fibrosis as assessed by AUROCs [2], [10], [13]–[16]. In multivariate analysis the ActiTest a biomarker of necrosis and inflammation add no supplementary prognostic value to the knowledge of FT and viral load. However more patients are needed to assess the independent prognostic value of ActiTest particularly in patients anti-HBe positive, at higher risk of flares.

#### Impact of HBV treatments

The results confirm the very significant impact of HBV treatments on repeated FT and AT, particularly in patients with moderate or severe fibrosis at baseline as previously observed in two other studies with paired samples [14], [16].

Our study was not conclusive for the use of FT-AT scores to determine the need for treatment, but the results supported a simple new definition of inactive HBV carrier. Because patients with normal FT-AT scores were unlikely to develop complications, decisions not to treat such patients were unlikely to be associated with clinical decompensation, at least over a relatively short follow-up period. The diagnostic and prognostic values of FT persisted among treated or non-treated patients.

In conclusion, FT has significant prognostic values at 4 years in patients infected by HBV, similar to that of liver biopsy. A combination of FT-AT and viral load more accurately defined the status of inactive HBV carrier than the ALT and viral load.
