# Correction: An Accurate Definition of the Status of Inactive Hepatitis B Virus Carrier by a Combination of Biomarkers (FibroTest-ActiTest) and Viral Load

**DOI:** 10.1371/annotation/9f8332b9-fdc0-48e8-967b-3c504024b9d1

**Published:** 2008-07-11

**Authors:** Yen Ngo, Yves Benhamou, Vincent Thibault, Patrick Ingiliz, Mona Munteanu, Pascal Lebray, Dominique Thabut, Rachel Morra, Djamila Messous, Frederic Charlotte, Françoise Imbert-Bismut, Dominique Bonnefont-Rousselot, Joseph Moussalli, Vlad Ratziu, Thierry Poynard

The twelfth author's name appears incorrectly. It should read: Dominique Bonnefont-Rousselot.

